# Fast fluorescence *in situ* hybridisation for the enhanced detection of *MET* in non-small cell lung cancer

**DOI:** 10.1371/journal.pone.0223926

**Published:** 2019-10-15

**Authors:** David Jonathan Duncan, Michel Erminio Vandenberghe, Marietta Louise Juanita Scott, Craig Barker

**Affiliations:** Precision Medicine, R&D Oncology, AstraZeneca, Cambridge, England, United Kingdom; University of Nebraska Medical Center, UNITED STATES

## Abstract

The c-Met/hepatocyte growth factor receptor pathway is frequently dysregulated in multiple cancer types, including non-small cell lung cancer (NSCLC). *MET* amplification has been shown to develop as a resistance mechanism to treatment in NSCLC. The identification of increased *MET* copy number within tumour cells is increasingly important to stratify those tumours and patients which are susceptible to treatment targetting MET kinase inhibition. Fluorescence *in situ* hybridisation (FISH) has been successfully employed to identify patients with abnormal *MET* gene copy number with numerous probes available for use. Here we report a FISH protocol that reduces probe hybridisation time in NSCLC tissue to 1 hour and compare the results with other protocols. *MET* gene copy number was determined in 20 NSCLC cases using 3 FISH probes: 1. Kreatech FISH, MET (7q31) SE 7 ready to use probes, hybridised using an overnight protocol; 2. Dako MET IQFISH probe with CEP7 ready to use probe, hybridised for 2 hours; 3. Kreatech MET (7q31) SE 7 XL FISH probe, prepared in SwiftFISH buffer and hybridised for 1 hour. The *MET* gene copy number and *MET*: centromere 7 gene ratio were determined for each tissue and cases categorised as having *MET* high or *MET* low status. All three FISH probes were shown to demonstrate good agreement with each other. Overall percentage agreement between probes was ≥90%. Intraclass correlation showed good agreement (ICC ≥0.80) between all three assays for *MET* gene copy number and *MET*: centromere 7 gene ratio. These FISH protocols provide evidence that rapid laboratory developed FISH assays with short turnaround time perform consistently with standard protocols, potentially enabling faster treatment decisions.

## Introduction

The c-Met/hepatocyte growth factor receptor (HGFR) pathway is one of the most frequently dysregulated pathways in human cancer. Receptor activation leads to the recruitment and activation of specific downstream signalling partners that participate in the regulation of diverse processes such as tumour cell growth, migration, scattering and metastasis. In the endothelial and stroma cells surrounding tumour tissue, the c-Met/HGFR pathway acts in a proangiogenic manner to stimulate cell proliferation, migration and survival, which can support tumour expansion and progression. Hepatocyte growth factor (HGF), the ligand for the receptor, and *MET* expression have been observed in tumour biopsies of most solid tumours and *MET* signalling has been documented in a wide range of human malignancies, including bladder, breast, cervical, colorectal, gastric, head and neck, liver, lung, ovarian, pancreatic, prostrate, renal and thyroid cancers, as well as in various sarcomas, haematopoietic malignancies and melanoma [1, 2]. Activating mutations in the tyrosine kinase domain of *MET* have been positively identified in patients with a hereditary form of papillary renal cancer, directly implicating *MET* in human tumourigenesis [3]. TCGA PanCancer Atlas studies and MSK-IMPACT Clinical Sequencing Cohort were queried for *MET* gene alterations using cBioPortal [4, 5]; irrespective of indication, the *MET* gene is altered in 3% of patients; 1 percent of patients were recorded with *MET* amplification. In Non Small Cell Lung cancer ~5% of queried patients showed a *MET* gene alteration, 1.66% of which were *MET* amplification. Mutation prevalence data from cBioPortal are presented in supporting information (S1 Fig. *MET* mutation prevalence per cancer type queried using cBioPortal). In several clinical studies, aberrant c-Met overexpression has been correlated with poor clinical outcome, rapid disease progression and short survival [6]. Overexpression of c-Met and HGF are also thought to result in resistance of tumour cells to chemotherapy and radiotherapy, correlating with development of distant metastases and shorter metastasis-free survival [2]. Furthermore, in addition to gene amplification or protein overexpression, enhanced signalling of the *MET* pathway can be induced by mutations resulting in exon 14 skipping [7]. Up to 22% of patients with non-small cell lung cancer (NSCLC) who progress on first-line EGFR-TKIs have *MET* amplification or other MET-based mechanisms of resistance [8–10]. *MET* amplification has been found after acquired resistance to EGFR tyrosine kinase inhibitors such as osimertinib [11].

*MET* amplification status in tissue biopsies can be determined using fluorescence *in situ* hybridisation (FISH), polymerase chain reaction (PCR) based technology and next generation sequencing (NGS) [6, 12]. FISH assays provide an easily accessible, reproducible solution for the enumeration of gene copy number [13] and are widely utilised in the clinical cytogenetic and oncology settings. Of particular note, *ALK* FISH and *HER2* FISH have been developed as companion diagnostic assays, to detect break apart genes and gene amplification respectively, exemplifying their use in clinical trials and for patient selection [14, 15]. FISH assays are of interest in conditions such as NSCLC cancer to detect *MET* gene amplification or aneuploidy, where the target may be drugable. Demonstrating fast turnaround time and accurate results across assay platforms is essential for FISH uptake in clinical trials and clinical practice.

FISH is a molecular cytogenetic technique used to identify specific segments of a chromosome by hybridising a fluorescently labelled probe to nucleic acids; the number of fluorescent signals is correlated with DNA copy-number [16, 17]. FISH assays typically exist in multiple formats; assays may be fully automated, partially automated, or manual [18–20]. Additionally, FISH assays have routinely been developed for use with formalin fixed paraffin embedded (FFPE) tissue, with specific protocols to prepare the tissue for probe hybridisation. Pretreatment methods balance the reversal of formalin fixation while maintaining tissue architecture and commonly combine heat, chemical and enzyme treatment [21, 22]. Probe vendors typically provide recommended pretreatment conditions, although some optimisation may be required. Clinical laboratories may wish to use a common pretreatment method for all probes in order to streamline testing logistics.

FISH probe hybridisation efficiency and therefore signal intensity and analysis are broadly dependent upon tissue pretreatment and hybridisation duration. The variability of probe hybridisation associated with reduced hybridisation time and pretreatment methods to our knowledge has not been extensively examined between vendors of *MET* FISH probes in NSCLC. Traditionally, FISH procedures require probe hybridisation to the target sequence for between 12–18 hours, usually carried out overnight. Recently, several groups have reported methods that reduce hybridisation time [19, 23].

Jorgensen *et al*. processed 159 gastro-intestinal cancer FFPE tissue samples using a *MET*/CEN-7 IQFISH Probe Mix (for investigational use only (IUO), Agilent Technologies, Glostrup, Denmark). Probes were hybridised to tissue using a short protocol requiring 90 minutes incubation. Samples were categorised as amplified or non amplified by two observers. The investigators showed 100% overall inter-reader agreement between cases using a short FISH protocol.

Furthermore, Tafe *et al*. compared two *HER2* FISH assays, the PathVysion HER2 DNA Probe Kit (Abbott, USA) and the HER2 IQ FISH pharmDx Kit (Agilent Technologies, Denmark), which require 14–18 hours and 90 minutes hybridisation and result in 2 day and 3–4 hour turnaround times respectively in breast and gastro-oesophageal cancer [24]. Cases were analysed in accordance with ASCO/CAP 2013 BR cancer guidelines [25]. Ten out of 30 cases were identified as amplified and the authors show 100% overall concordance between both assays.

Fast assay turnaround time is important for patient care where assay results define treatment. Reducing FISH assay turnaround time to within 1 day therefore allows patients to access treatment more rapidly. Here, we evaluate the robustness of fast FISH protocols for the detection of *MET* amplification in NSCLC and reduce probe hybridisation time to as little as 60 minutes, thus shortening assay duration further compared to previously published assays while maintining assay performance.

## Materials and methods

### Tissue

Twenty commercial NSCLC samples (TriStar Technology Group LLC, Washington, US and Trans-Hit Biomarkers, Canada) were selected to represent the same proportion of patients with *MET* amplification as seen in NSCLC patients with tyrosine kinase inhibitor resistance. Consecutive tissue sections were cut at 4μm thickness. Tissue sections were probed using FISH assays within 3 months of tissue sectioning [26].

### FISH assays

*MET* gene copy number was determined using 3 FISH protocols (summarised in Table 1). A common tissue pretreatment protocol was used for all tissue prior to probe hybridisation, in order to ensure that any differences between the results of the FISH protocols was not due to variability in pre-treatment conditions. Tissue sections were dewaxed, treated with BOND epitope retrieval solution 2 for 25 minutes at 97°C followed by incubation with BOND enzyme (diluted to a final concentration of 1:3000) for 25 minutes at 37°C using the Leica BOND Rx instrument (Leica Microsystems Inc., Buffalo Grove, IL). Following pretreatment, tissue sections were removed from the instrument. A single section from each case was then subjected to one of three FISH protocols summarised below (Table 1). The pretreatment protocol differed to manufacturers recommendations; stringency wash conditions however, as recommended in each manufacturers instructions were used to remove excess probe from tissue prior to counterstaining [27–30].

**Table 1:** Summary of FISH protocols.

FISH probe	Abbreviated protocol name	Probe Denaturation		Probe Hybridisation		Stringency wash	
		Temperature (°C)	Time (min)	Temperature (°C)	Time (hours)	Temperature (°C)	Time (min)
**Kreatech^™^ FISH probes for MET (7q31) and SE 7 (ready to use)**	**Standard Kreatech assay**	80	5	37	12–18	72	1
**Dako MET IQFISH probe with CEP7 (ready to use)**	**Fast Dako assay**	80	10	45	2	63	10
**Kreatech^™^ MET (7q31) SE 7 XL FISH probe for BOND (diluted in SwiftFISH hybridisation buffer).**	**Fast Kreatech assay**	83	5	43	1	72	1

A single section from each case was probed with Kreatech FISH probes for MET (7q31) and SE 7 (Leica Biosystems, Amsterdam) using a ThermoBrite system. The probe is premixed in ready to use format. The Kreatech FISH, MET (7q31) SE 7 probe was denatured for 5 minutes at 80°C followed by 12–16 hours hybridisation at 37°C. Tissue was washed under high stringency conditions (72°C) for 1 minute with 0.4x SSC/ 0.3% Igepal (according to Leica Biosystems Tissue Digestion Kit 1 [30]). Tissue was then transferred to 2x SSC/ 0.1% Igepal for 2 minutes at room temperature. This assay is referred to from here as the standard Kreatech assay.

A second consecutive section from each case was probed with Dako MET IQFISH probe with CEP7 (Agilent Technologies, UK) according to the manufacturers instructions [27]. Using a ThermoBrite system the probe was denatured at 80°C for 10 minutes followed by hybridisation at 45°C for 2 hours. The probe is premixed in ready to use format. Following hybridisation tissue was washed under high stringency conditions in 1X SSC/ Tween 20 for 10 minutes at 63°C, followed by incubation for 6 minutes at room temperature in 1x Tris HCl (from Dako Histology FISH Accessory Kit, Agilent Technologies, UK). This assay is referred to from here as the fast Dako assay.

A third consecutive section from each case was also probed using Kreatech MET (7q31) SE 7 XL FISH probe for BOND, supplied in concentrate and diluted to 1x working concentration in SwiftFISH rapid hybridisation buffer (Empire Genomics, New York, USA). Using a ThermoBrite system the probe was denatured at 83°C for 5 minutes followed by hybridisation for 60 minutes at 43°C (according to manufacturers instructions [31]). Tissue was washed under high stringency conditions (72°C) for 1 minute with 0.4x SSC/ 0.3% Igepal (from Leica Biosystems Tissue Digestion Kit 1). Tissue was then transferred to 2x SSC/ 0.1% Igepal for 2 minutes at room temperature. This assay is referred to from here as the fast Kreatech assay.

Following completion of each protocol desribed above, tissue sections were dehydrated rapidly in alcohol then air dried in the dark. Tissue was counterstained using VECTASHIELD Mounting Medium (Vector Laboratories, UK) with 4',6-Diamidino-2-Phenylindole, Dihydrochloride (DAPI).

### Tissue analysis

Tissue FISH signals were assessed consistently with guidelines described previously for tissue processing, tumour identification and signal enumeration for the assessment of EGFR FISH [32]. Tissue sections were imaged using a Zeiss Axio Scan.Z1 scanner for fluorescence and brightfield (Carl Zeiss Ltd, UK) using identical scanning parameters. Tissue was scanned and light detected at wavelengths appropriate for each fluorophore. Tissue was excited at 353, 493 and 548 nm for 19.31, 250 and 370 ms respectively and emitted light detected at 465, 517 and 561 nm for DAPI, green and orange/red fluorescence. For all probe sets, the probe specific to 7q31 (*MET*) was labelled with an orange/red fluorophore and the probe specific to centromere 7 was labelled with a green fluorophore. Four z stack images were taken at 1 μm intervals and amalgamated using Zeiss extended depth of focus (EDF) feature which combines regions of greatest contrast within the Z stack image to maximise the contrast of the final image.

Following digitisation, tumour area was identified and tissue assessed to ensure FISH signals were clear and visible. Using low magnification, the entire tumour area was visually evaluated to identify any regions of signal amplification or aneuploidy. The number of *MET* and centromere 7 signals were manually enumerated in 50 tumour cell nuclei at x40 magnification. Nuclei with the highest number of FISH signals per field of view were enumerated. The gene copy number and *MET*:centromere 7 ratio were calculated in 50 tumour cell nuclei per case and the mean score recorded. If the average *MET* gene copy number was ≥5 or the *MET*:centromere 7 ratio was ≥2, the tissue section was determined to show *MET* high gene status in accordance with parameters used previously to enroll patients to clinical trials [33].

### Statistical analysis

The overall agreement of the classification of the cases for each assay was calculated against the standard Kreatech assay (which requires overnight hybridisation) as the reference assay. Bland Altman plots were used to visualise agreement in individual scores with the reference assay [34]. The agreement interval of the difference between each assay were calculated as ± 2 standard deviations (±2 SD) of the mean. Intraclass correlation coefficients (ICC 2,1) were calculated using the psych R package (R Foundation for Statistical Computing) to assess the reliability between tests [35].

## Results

Twenty commercial NSCLC cases were assessed with each of three FISH assays. All 20 cases were successfully analysed when tested with the standard Kreatech and fast Dako assays. Seventeen cases were successfully tested with the fast Kreatech assay. Three cases were not enumerated due to the absence of clear interpretable FISH signals.

### *MET* status and inter-assay reproducibility

**Fig 1:**
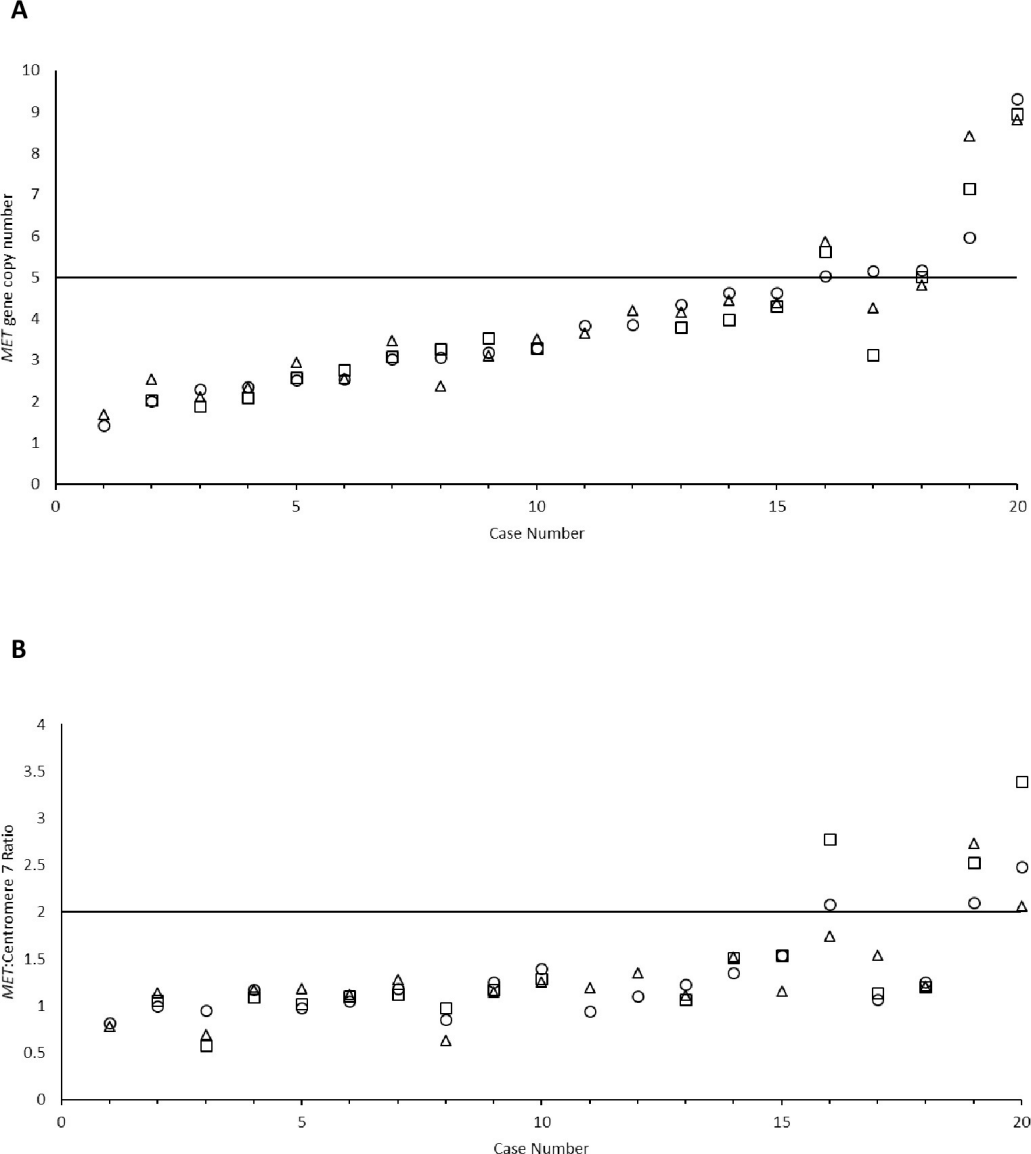
***MET* gene copy number (A) and *MET*: Centromere 7 ratio (B) of cases probed with the standard Kreatech assay (circles), fast Dako assay (triangles) and fast Kreatech assay (squares).** Horizontal line represents the threshold to determine *MET* high or *MET* low status. Overall *MET* status showed discordance for cases 17 and 18. Although *MET*: Centromere ratio of case 16 falls either side of the cut off, the *MET* gene copy number status for this case was high using all three assays. Cases 1, 11 and 12 were not evaluated using the fast Kreatech assay.

Using the standard Kreatech assay, 5 of the 20 specimens tested showed *MET* high status. Three out of five cases were also *MET* high using the fast Dako assay. Of the seventeen cases successfully tested with the fast Kreatech assay, 5 were determined as *MET* high using the standard Kreatech assay, four were determined as *MET* high using the fast Kreatech assay (Fig 1, Table 2). Average *MET* gene copy number and average *MET*: centromere 7 gene copy number ratio of all cases are presented in supporting information (S1 Table. *MET* gene copy number and *MET*: centromere 7 gene ratio of cases tested using the standard Kreatech, fast Dako and fast Kreatech assays).

**Table 2:** Number of cases determined as *MET* high or *MET* low using fast Dako and fast Kreatech assays compared to the standard Kreatech assay.

	Standard Kreatech *MET* high	Standard Kreatech *MET* low	Total
Fast Dako *MET* high	3	0	3
Fast Dako *MET* low	2	15	17
Total	5	15	20
Fast Kreatech *MET* high	4	0	4
Fast Kreatech *MET* low	1	12	13
Total	5	12	17

20 cases were tested using standard Kreatech and fast Dako assays, 17 cases were evaluated using the fast Kreatech assay.

**Fig 2:**
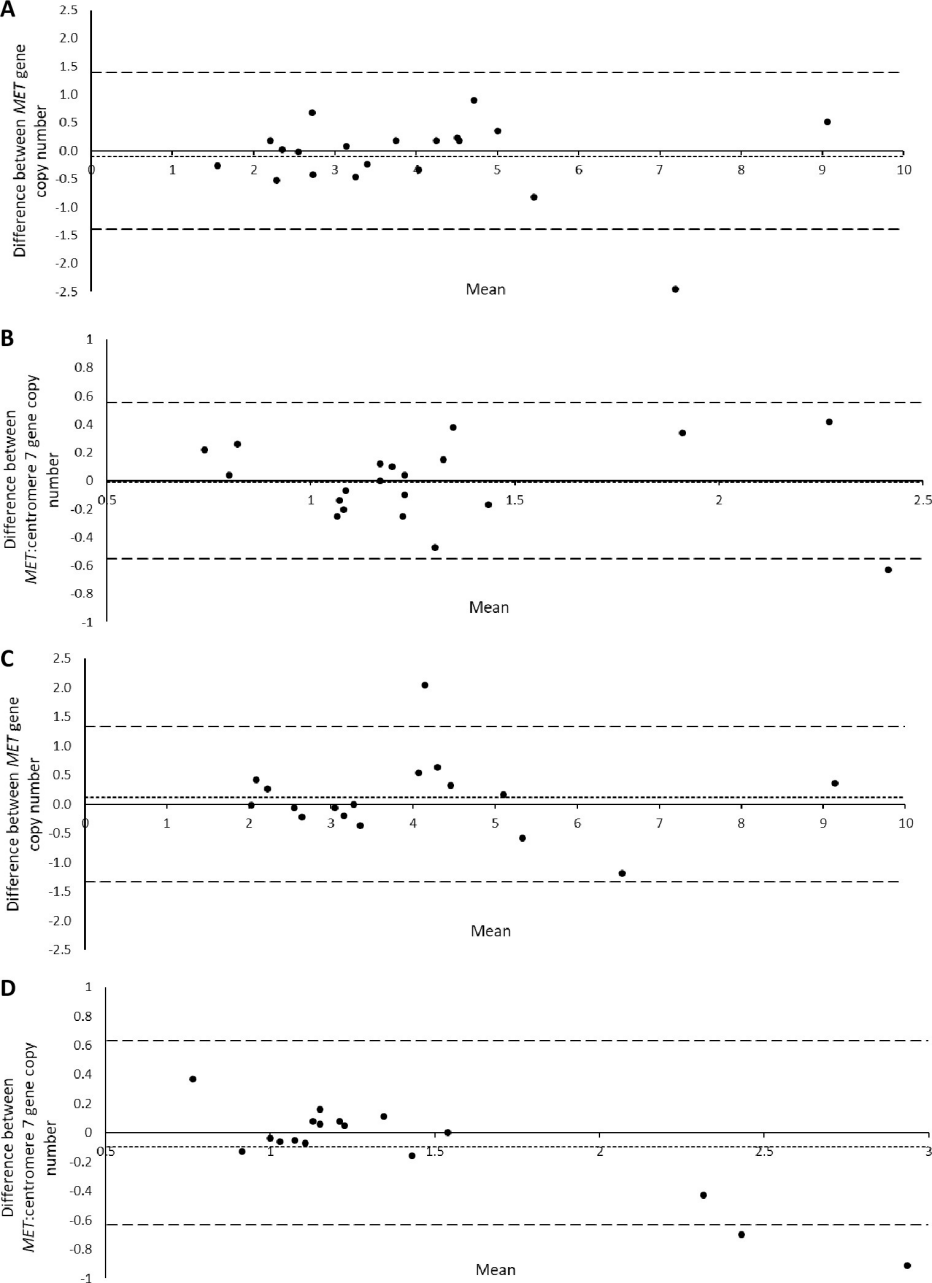
**Bland altman plot of inter-assay agreement of *MET* gene copy number and *MET*: centromere 7 gene ratio between the standard Kreatech and fast Dako assay (A and B) and between the standard Kreatech assay and the fast Kreatech assay (C, D).** Short dashed line represents the mean of the difference between assays, long dashed lines indicate ±2 standard deviations (±2 SD).

Agreement between assays was assessed using Bland Altman plots. Fast Dako and fast Kreatech assays were compared to the standard Kreatech assay using *MET* gene copy number and *MET*: centromere 7 gene ratio (Fig 2). The mean of the difference between each assay was calculated. For *MET* gene copy number, the mean difference between the standard Kreatech assay and fast Dako assay was -0.10 and the mean difference for *MET*: centromere 7 gene ratio was -0.01. For *MET* gene copy number, the mean difference between the standard Kreatech assay and fast Kreatech assay was 0.12 and the mean difference for *MET*: centromere 7 gene ratio was -0.10.

**Fig 3:**
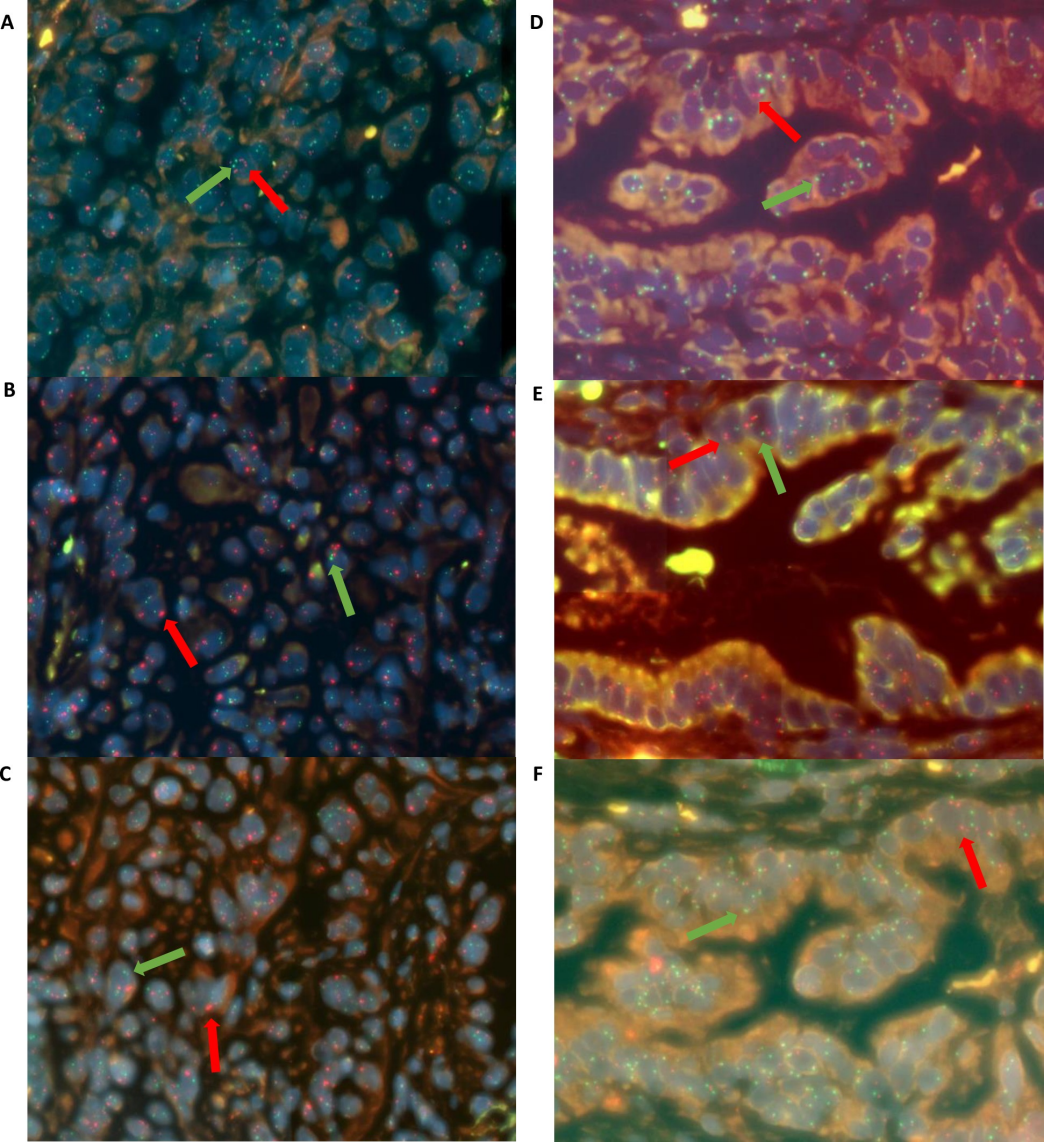
Probe hybridisation to NSCLC cases showing marginally increased and normal *MET* gene copy number. Probe hybridisation efficiency was reproducible between the Standard Kreatech assay (A, D), fast Dako assay (B, E) and fast Kreatech assays (C, F). Tissue with increased (A, B, C) and normal gene copy number (D, E, F) are shown. Red fluorophore is conjugated to probe specific to *MET* gene (specific examples highlighted with red arrows), green fluorophore is conjugated to probe specific for chromosome 7 centromere (highlighted with green arrows).

The agreement interval (±2 SD) of the standard Kreatech assay and fast Dako assay was ±1.39 for *MET* gene copy number and ±0.55 for the *MET*: centromere 7 gene ratio. The agreement interval (±2 SD) between the standard Kreatech probe and the fast Kreatech probe was ±1.33 for *MET* gene copy number and ±0.63 for *MET*: centromere 7 gene ratio. ICC showed good agreement between all three assays for *MET* gene copy number (ICC 2,1 = 0.94, 95% CI 0.88–0.97) and *MET*: centromere 7 gene ratio (ICC 2,1 = 0.80, 95% CI 0.63–0.91). Representative images of probe hybridisation to tissue with marginally increased and normal average *MET* gene coopy number are shown in Fig 3.

Overall percentage agreement (OPA) in classification of cases into *MET* high and *MET* low was calculated using the standard Kreatech assay as reference. OPA ≥90% was observed between the standard Kreatech assay and the fast Dako assay and between the standard Kreatech assay and the fast Kreatech assay (Table 3).

**Table 3:** Overall percentage agreement (OPA) between FISH assays.

Assay	Dako MET IQFISH probe with CEP7 (Fast Dako assay)	Kreatech^™^ MET (7q31) SE 7 XL FISH probe with SwiftFISH buffer (Fast Kreatech assay)
	OPA (lower 95% CI), %	OPA (lower 95% CI), %
**Kreatech^™^ FISH MET (7q31) and SE 7 probes (Standard Kreatech assay)**	90 (68)	94 (71)

The standard Kreatech assay was used as a reference assay to determine the OPA with the fast Dako assay or the fast Kreatech assay. Lower 95% confidence intervals are also presented.

## Discussion

Recently, FISH protocols have emerged which utilise shorter processing time, through a reduction of hybridisation duration [23, 24]. These rapid assays typically take advantage of different probe hybridisation buffers which allow more efficient probe hybridisation [36]. Here, we report a *MET* FISH protocol that reduces probe hybridisation time in NSCLC tissue to 1 hour and compare the results with other protocols. We assess the agreement between three FISH probes by measuring the average *MET* gene copy number and average *MET*: centromere 7 gene ratio in a cohort of commercial NSCLC cases. We successfully demonstrate equivalence between assays and maintain high quality staining, using just 1 or 2 hours hybridisation, enabling FISH assays to be completed within a standard 7.5 hour laboratory shift.

FISH signals were enumerated in 20 commercial NSCLC FFPE cases probed with a standard Kreatech assay and a fast Dako assay. Seventeen out of 20 commercial cases probed using the fast Kreatech assay were successfully analysed; 3 cases were void of signal or showed weak signal following the fast Kreatech assay and were not analysed. A universal pretreatment procedure using the Leica BOND Rx instrument was employed prior to application of FISH probes in this study. Cases void of FISH signal were not retested with adapted pretreatment conditions. The practice of marginally adjusting tissue pretreatment is common during FFPE tissue processing prior to FISH probe hybridisation [21]. Universal pretreatment ensured any variability between assays was not confounded by the pretreatment step, it does however limit the opportunity to further optimise the assays; possibly improving probe hybridisation efficiency and potentially increasing the fast Kreatech assay pass rate to be more in line with the standard Kreatech assay and fast Dako assay [13]. Additionally, a relatively small sample size was tested in this study, limiting our ability to precisely elucidate exactly why 3 cases were void of FISH signals using the fast Kreatech assay. These data are however sufficient to demonstrate the feasibility of a previously untested fast assay; further expanded sample sets and validation studies should be performed to comprehensively determine fast assay reproducibility.

The effectiveness of tissue pretreatment prior to probe hybridisation may also be affected by pre analytical tissue processing, such as fixation. All commercial cases were FFPE tissue, the fixation time however was not specified beyond routine processing. Coupled with short hybridisation times, pre analytical variation of tissue processing may have led to the absence of successful probing in 3 cases using the fast Kreatech assay.

We have demonstrated good correlation between three FISH probes; for *MET* copy number ICC = 0.94 and for average *MET*: centromere 7 ratio ICC = 0.80. The mean difference between the standard Kreatech assay and the fast Dako assay or fast Kreatech assay showed no systematic differences between assays (Fig 2). The variability observed in the cases outside ±2 SD may be due to the low amplification of gene copy number status of the cases, which may be heterogenous within the tissue and therfore more difficult to count than highly amplified or diploid cases. The probes used here are of similar size (~400 KB) and have been validated to hybridise to 7q31. These data illustrate that different FISH probes and assay protocols could potentially be utilised interchangeably to determine *MET* gene status, provided the probes and assays are fully validated.

In this study, a single analyst enumerated all cases. Inter- and intra-laboratory reproducibility have been examined using signal enumeration FISH probes for the identification of gene amplification and increased gene copy number. These previous studies examined inter- and intra-laboratory reproducibility of a single *HER2* FISH assay [19]. The inter-laboratory reproducibility of the assay was investigated across three sites using 11 FFPE breast cancer specimens. Consecutive sections from each case were probed and analysed at least 5 times by a single blinded observer at each site on non consecutive days. Inter-laboratory reproducibility coefficient of variation was shown to be 11.6%. The authors also observed 97.1% overall agreement of inter-laboratory reproducibility. Lot-to-lot and day-to-day variation showed the total coefficient of variation in the study was 4.3% (95% CI 3.7; 4.9). Further investigation is required to determine the extent of inter-reader variability of *MET* FISH assays. Concordance between readers will be influenced by a standardised approach to nuclei and signal identification in addition to sufficient training in relation to the enumeration strategy [32, 37].

*MET* high status was defined here as average *MET* gene copy number ≥5 or *MET*: centromere 7 ratio ≥2. OPA between assays, using the Kreatech standard assay as a reference assay was ≥90%. Lower 95% CI were 68% for the fast Dako assay compared to the standard Kreatech assay and 71% for the fast Kreatech assay compared to the standard Kreatech assay. The low CI of agreement was driven by two cases which showed *MET* high status using the standard Kreatech assay and *MET* low status using the fast Dako assay (Fig 1 cases 17 and 18). The average *MET* gene copy number of these two cases were determined as 4.26 and 4.82 using the fast Dako assay and 5.16 and 5.18 respectively using the standard Kreatech assay. The difference of average gene copy number between these cases is <1 and falls across the cut off defined for this study. Cases such as these represent “borderline” or “equivocal” cases. Both these cases lie close to the threshold for determining *MET* high or low status. In order to gain a clearer understanding of the gene copy number, enumeration of additional nuclei may have been required. Furthermore, borderline or equivocal cases could be analysed by more than one reader in order to gain a consensus opinion as to the gene amplification observed, this is particularly important for diagnostic cases where treatment decisions are made.

Additionally, difficult to analyse cases such as these may harbour genetic heterogeneity. Jørgensen *et al* postulate that a heterogenous signal pattern is associated with *MET* amplification in gastroesophageal tumour specimens, showing 12 out of 159 tumour samples tested exhibited heterogeneous signal distribution, eight (66%) out of the 12 specimens were *MET* amplified; *MET* amplification in this instance was defined as *MET* centromere 7 gene ratio ≥2.0 by these authors [23].

Atypical FISH signal patterns have been observed in other pathologies such as *HER2* [38]. These authors observed that approximately 10% of ISH results from a single centre showed an unusual signal pattern. Some parallels may be expected in *MET* ISH and atypical signal patterns will in future require consistent reporting.

Cases in this study were imaged using a Zeiss Axio Scan.Z1 slide scanner. Z stack images were captured and amalgamated using Zeiss EDF to visualise the maximum projected signal intensity from each Z stack image, thus preventing the perception that FISH signals may have diminished. FISH signals in 50 nuclei of each case were enumerated manually. This approach has been commonly used for signal enumeration and tissue analysis in various FISH probe companion diagnostic kits (and is described in manufacturers instructions). In order to be reproducible between readers and laboratories, clear guidelines and standard instructions for tissue preparation and signal enumeration are required. For example, clear guidelines for the preparation of samples and analysis of *HER2* and *ALK* FISH assays is regularly reviewed and updated [39, 40]. Furthermore independent quality assurance schemes will enable consistent reporting.

In addition to clear analysis guidelines, automated image analysis could aid in the determination of *MET* status. Numerous solutions for automated enumeration of FISH signals in a clinical environment have been developed for probes such as *HER2* [18, 41, 42]. Given the similarities between *HER2* and *MET* FISH analysis it is conceivable an automated image analysis solution for *MET* FISH could be developed further for routine diagnostic use. Furthermore, machine learning could potentially be developed to fully automate tumour cell identification and FISH signal enumeration. Combining image analysis with a fast FISH assay could offer diagnostic testing laboratories significant resource savings in addition to reduced turnaround time. Moreover, image analysis platforms may aid analytical reproducibility and inter-laboratory precision in a clinical environment.

Fast assays may become more important to testing laboratories as treatment decisions are required more quickly and demands on limited resources grow; a larger study to provide evidence FISH assays are interchangeable is required. Different specimen types such as cell blocks derived from fine needle aspirates or needle biopsies were not included here. Fine needle aspirates are a source of biopsy for NSCLC diagnosis [43] and an examination of this sample type, particularly aspirates prepared into FFPE cell blocks, is required to further understand the utility of fast FISH assays in a clinical diagnostic setting. Whether these sample types yield sufficient tissue for diagnosis and are indicative of response to a specific treatment remains to be validated.

In primary or metastatic NSCLC, if resection or surgery is not a component of treatment, tissue samples may be difficult to obtain. Additionally, repeated biopsy in a patient monitoring setting may not be clinically feasible. In these scenarios alternative sample types to FFPE tissue for biomarker identification such as circulating tumour cells (CTCs), may be an alternative [44]. *ALK* gene rearrangements and copy number gains have been detected by FISH on CTCs [45] and the evolution of the number of cells with *ALK* copy number gains was shown to be associated with progression free survival and early progression. The authors present these findings as a potential strategy for real time monitoring of patient response using FISH. Additionally, a method has been developed to capture CTCs based on c-Met overexpression [46]. *MET* FISH status of a small number of patient CTCs in this study were successfully assessed confirming the presence of gene amplification, although the use of such an assay in a clinical setting is yet to be evaluated. Rapid FISH protocols may therefore be applied to a number of alternative sample types and may provide predictive or prognostic information under these circumstances. Further investigation of these sample types using a validated assay is required.

Feasibility data presented here demonstrate good agreement between three *MET* FISH probes utilising overnight, 2 and 1 hour hybridisation times. All tissue was pretreated using a common automated protocol, followed by manual probe application. OPA between assays was shown to be greater than 90%. Probe hybridisation for 1 or 2 hours offers testing laboratories the opportunity to significantly reduce turnaround time potentially enabling patients faster access to treatment decisions. Probes from different manufacturers may be used interchangeably, enabling consistent results, provided protocols are fully validated. Full automation and image analysis could potentially provide even faster treatment decisions and reduce assay variability, increasing diagnostic accuracy.

## Supporting information

S1 Fig *MET* mutation prevalence per cancer type queried using cBioPortal. (DOCX)

S1 Table *MET* gene copy number and *MET*: Centromere 7 gene ratio of cases tested using the standard Kreatech, fast Dako and fast Kreatech assays. (DOCX)
