# Supplementary material for: Fast fluorescence in situ hybridisation for the enhanced detection of MET in non-small cell lung cancer
Source: PLoS One. 2019 Oct 15;14(10):e0223926. doi: 10.1371/journal.pone.0223926 (PMC6793848; doi:10.1371/journal.pone.0223926)
Supplement: S1 Fig — (DOCX) [file pone.0223926.s001.docx]

**S1. Fig. *MET* mutation prevalence per cancer type queried using cBioPortal**. Full query details via link: <http://bit.ly/2Ohh7wc>

**
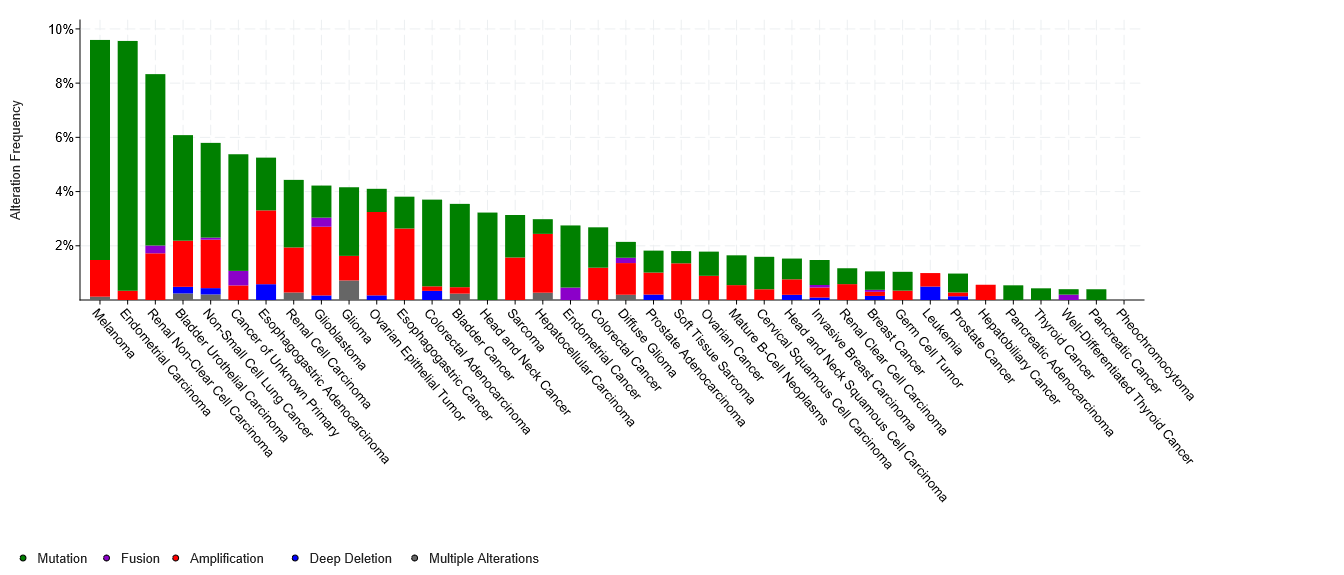
**
