# Supplementary material for: Fast fluorescence in situ hybridisation for the enhanced detection of MET in non-small cell lung cancer
Source: PLoS One. 2019 Oct 15;14(10):e0223926. doi: 10.1371/journal.pone.0223926 (PMC6793848; doi:10.1371/journal.pone.0223926)
Supplement: S1 Table — (DOCX) [file pone.0223926.s002.docx]

**S1. Table. *MET* gene copy number (GCN) and *MET*: centromere 7 gene ratio of cases tested using the standard Kreatech, fast Dako and fast Kreatech assays**

| **Case number** | **Standard Kreatech assay** | | **Fast Dako assay** | | **Fast Kreatech assay** | |
| --- | --- | --- | --- | --- | --- | --- |
|  | ***MET* GCN** | ***MET*: centromere 7 gene ratio** | ***MET* GCN** | ***MET*: centromere 7 gene ratio** | ***MET* GCN** | ***MET*: centromere 7 gene ratio** |
| **1** | 1.42 | 0.82 | 1.68 | 0.78 | Weak signal not evaluable | |
| **2** | 2.02 | 1 | 2.54 | 1.14 | 2.04 | 1.06 |
| **3** | 2.3 | 0.95 | 2.12 | 0.69 | 1.88 | 0.58 |
| **4** | 2.36 | 1.17 | 2.34 | 1.17 | 2.1 | 1.09 |
| **5** | 2.52 | 0.98 | 2.94 | 1.18 | 2.58 | 1.02 |
| **6** | 2.54 | 1.05 | 2.56 | 1.12 | 2.76 | 1.1 |
| **7** | 3.02 | 1.18 | 3.48 | 1.28 | 3.08 | 1.12 |
| **8** | 3.06 | 0.85 | 2.38 | 0.63 | 3.26 | 0.98 |
| **9** | 3.18 | 1.25 | 3.1 | 1.15 | 3.54 | 1.17 |
| **10** | 3.28 | 1.4 | 3.52 | 1.25 | 3.28 | 1.29 |
| **11** | 3.84 | 0.94 | 3.66 | 1.19 | Weak signal not evaluable | |
| **12** | 3.86 | 1.1 | 4.2 | 1.35 | Weak signal not evaluable | |
| **13** | 4.34 | 1.23 | 4.16 | 1.11 | 3.8 | 1.07 |
| **14** | 4.62 | 1.35 | 4.44 | 1.52 | 3.98 | 1.51 |
| **15** | 4.62 | 1.54 | 4.39 | 1.16 | 4.3 | 1.54 |
| **16** | 5.04 | 2.08 | 5.86 | 1.74 | 5.62 | 2.78 |
| **17** | 5.16 | 1.07 | 4.26 | 1.54 | 3.12 | 1.14 |
| **18** | 5.18 | 1.25 | 4.82 | 1.21 | 5.02 | 1.2 |
| **19** | 5.96 | 2.1 | 8.42 | 2.73 | 7.14 | 2.53 |
| **20** | 9.32 | 2.48 | 8.8 | 2.06 | 8.96 | 3.39 |
